# Changing Landscape of Chronic Liver Diseases in a Tertiary Level Hospital of Bangladesh Over the Last 10 Years

**DOI:** 10.1155/ijh/5525981

**Published:** 2026-04-28

**Authors:** Md. Hasan Shahriar, Avizit Sarker, Tapu Ghosh, Md. Enamol Hoque, Md. Samsul Arafin, Md. Tanveer Rahman, Md. Shahinul Alam

**Affiliations:** ^1^ Department of Hepatology, Bangladesh Medical University, Dhaka, Bangladesh; ^2^ Department of Microbiology, Dhaka Medical College, Dhaka, Bangladesh, dmc.edu.bd; ^3^ Department of Gastroenterology and Hepatology, Monno Medical College and Hospital, Manikganj, Bangladesh; ^4^ Department of Hepatology, Sir Salimullah Medical College and Mitford Hospital, Dhaka, Bangladesh; ^5^ Department of Hepatology, Sher-E-Bangla Medical College Hospital, Barishal, Bangladesh; ^6^ Department of Hepatology, Rajshahi Medical College Hospital, Rajshahi, Bangladesh

**Keywords:** etiology, HBV, HCV, last 10 years, NAFLD/NASH, tertiary level hospital

## Abstract

Given significant advances in the treatment of viral hepatitis and the growing epidemic of obesity, the burden of the different types of chronic liver diseases in Bangladesh may be changing. Our aim was to assess the shift in the prevalence of different chronic liver disease etiologies in a tertiary level hospital of Bangladesh over the last 10 years. This was a retrospective observational study conducted in the Department of Hepatology in Bangabandhu Sheikh Mujib Medical University (BSMMU), Dhaka, Bangladesh. It was based on data from the hospital records (2013–2016 and 2017–2022). A total of 4658 patients were included from the hospital registry between 2013 and 2022. The etiologies of chronic liver disease were compared between two time periods: (2013–2016) and (2017–2022) among these patients. A significant decrease in the prevalence of chronic hepatitis B from 51.1% (2013–2016) to 44.4% (2017–2022) (*p* < 0.001), chronic hepatitis C from 11.3% (2013–2016) to 10.4% (2017–2022) (*p* = 0.032), and non‐B–non‐C from 11.7% (2013–2016) to 8.6% (2017–2022) (*p* < 0.001) was observed. In contrast, the prevalence of nonalcoholic fatty liver disease (NAFLD/NASH) increased from 1.2% (2013–2016) to 8.1% (2017–2022) (*p* < 0.001); anti‐HBc (total) from 3.2% (2013–2016) to 5.1% (2017–2022) (*p* = 0.001), and autoimmune hepatitis (AIH) from 0.2% (2013–2016) to 0.4% (2017–2022) (*p* = 0.038) also showed a significant increase. Over the last decade (2013–2022), NAFLD has emerged as a rapidly increasing cause of chronic liver disease in Bangladesh, whereas viral etiologies and AIH show a declining trend. Policy makers, clinicians, and stakeholders should take attention to recognize the situation and act properly.

## 1. Introduction

Chronic liver disease (CLD) is characterized by a progressive deterioration of liver function—such as synthesis of clotting factors and other proteins, detoxification of harmful metabolites, and excretion of bile—over a period exceeding 6 months [1]. Liver diseases are responsible for approximately 2 million deaths annually worldwide, of which 1 million are due to complications and sequelae of liver cirrhosis, and 1 million are due to viral hepatitis and hepatocellular carcinoma (HCC) [2]. According to the latest data from the World Health Organization (WHO) in 2020, death due to liver disease is the ninth leading cause of overall death in Bangladesh (21,024 people or 2.94%) [3]. Traditionally, alcoholism and viral hepatitis (particularly hepatitis B and C) have been the most common causes of cirrhosis around the world. However, in recent years, changes have been observed in the etiology of liver cirrhosis [2]. The leading cause of cirrhosis worldwide is now nonalcoholic fatty liver disease (NAFLD), which is associated with obesity, insulin resistance, and metabolic syndrome [4]. Similarly, the etiology of CLD is changing in Bangladesh. Historically, viral hepatitis B and C have been the leading causes of CLD in the country. With effective vaccination and treatment strategies, their prevalence is now decreasing. On the contrary, with the increasing prevalence of obesity, diabetes, and a sedentary lifestyle, NAFLD and nonalcoholic steatohepatitis (NASH) are becoming more prevalent in Bangladesh [5].

Approximately 60% of the world′s population reside where hepatitis B virus (HBV) is highly endemic; consequently, 15%–40% of these individuals will have serious sequelae such as cirrhosis or HCC [6]. Bangladesh is a country of intermediate risk, where 5.3 deaths per 100,000 population occurs due to HBV infections. Hepatitis C virus (HCV) accounts for 30% cases of cirrhosis and 17% cases of HCC in Bangladesh. Previous studies have reported a prevalence ranging from 0.2% to 1% in general population [7].

In Asia, prevalence of NAFLD has been found in the range of 15%–30% in the general population and over 50% in patients with DM and metabolic syndrome [8]. The overall prevalence of NAFLD in Bangladesh was 33.86%. Among the NAFLD patients, the rate of NASH was found to be 42.4%. Higher prevalence of NAFLD has been reported among females residing in rural areas and middle‐aged adults (45–54 years). Individuals with diabetes and hypertension were at a higher risk of having NAFLD. The odds of having NAFLD were 4.51 and 10.71 times higher among overweight and obese participants, respectively, as compared with normal‐weight participants [5].

Prevalence of anti‐HBc is high in the HBV‐endemic area. The prevalence of anti‐HBc in Bangladesh among the general population is 31% and 39.3% among CKD patients [9]. Autoimmune hepatitis (AIH) is more common in Europe and the number is increasing. Female to male ratio is 4:1. It is rare in Asia where the disease is usually detected at an advanced age with higher mortality [2].

HCC is the most common primary malignant tumor of the liver [6]. The prevalence of HCC in Bangladesh is 35% of all liver diseases [10]. HBV is associated with 61% cases of HCC. [11] and HCV for 6% of patients in Bangladesh [12]. The most common risk factor and predisposing condition worldwide is chronic infection with the HBV, which accounts for 52.3% of all HCC. Cirrhosis is the most important additional risk factor for HCC [13].

In this study, we intended to assess recent changes in the prevalence of different CLDs over the last 10 years in a tertiary level hospital of Bangladesh.

## 2. Materials and Methods

### 2.1. Ethics Statement

Ethical clearance for the study was taken from the Institutional Review Board (IRB) of Bangabandhu Sheikh Mujib Medical University (BSMMU), Dhaka, Bangladesh prior to the commencement of this study.

### 2.2. Study Participants

This was a retrospective observational study. This study was based on data obtained solely from the hospital registry in the department of Hepatology, BSMMU, Dhaka, Bangladesh. All adult (≥ 18 years of age) hospital‐admitted patients with archival evidence of CLD from 2013 to 2022 were included. The major categories of CLD included in this study were: (a) compensated cirrhosis of the liver due to any cause mainly HBV, HCV, NASH, alcohol, Wilson′s disease, AIH, non‐B–non‐C (NBNC), cryptogenic, and so on; (b) decompensated cirrhosis of the liver by the causes mentioned above; (c) cirrhosis of the liver with HCC; (d) acute‐on‐chronic liver failure (ACLF); and (e) recompensated cirrhosis of the liver. Exclusion criteria were patients diagnosed with acute viral hepatitis, acute liver failure, and HCC without cirrhosis of the liver. Patients with data insufficient for ruling in or ruling out CLD were excluded from the study.

**Figure 1:**
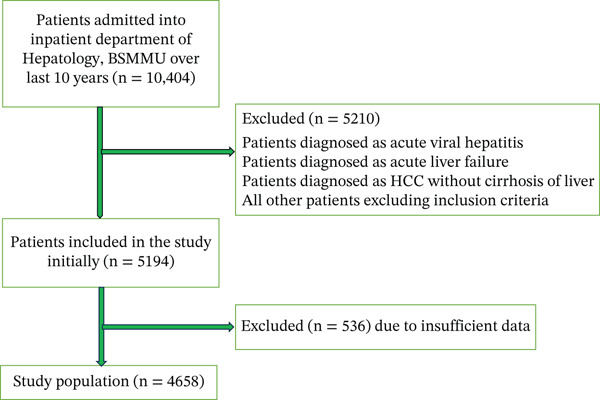
Flow chart of patient selection for the study.

A total of 10,404 patients were admitted into the Inpatient Department of Hepatology, BSMMU over the last 10 years (2013–2022). Among these patients, 5210 patients were initially excluded according to exclusion criteria and other patients outside the inclusion criteria. From the remaining 5194 patients, 536 patients were excluded further due to insufficient data (Figure 1).

The operational definition of “recompensation” requires fulfillment of all the following criteria:

a. Removal/suppression/cure of the primary etiology of cirrhosis (viral elimination for hepatitis C, sustained viral suppression for hepatitis B, and sustained alcohol abstinence for alcohol‐induced cirrhosis);
b. Resolution of ascites (off diuretics), encephalopathy (off lactulose/rifaximin), and absence of recurrent variceal hemorrhage (for at least 12 months);
c. Stable improvement of liver function tests (albumin, INR, and bilirubin) [14].

ACLF is defined as an acute insult to the liver manifesting as jaundice (serum bilirubin ≥ 5 mg/dL (85 *μ*mol/L) and coagulopathy (INR ≥ 1.5 or prothrombin activity < 40%) complicated within 4 weeks by clinical ascites and/or encephalopathy in a patient with previously diagnosed or undiagnosed CLD/cirrhosis, and is associated with a high 28‐day mortality [15].

### 2.3. Statistical Analysis

Data from all eligible patients were entered into SPSS software for statistical analysis to find out the exact percentage of CLD of different etiology and disposal of each patient to find out the mortality rate. The percentage of various demographic characteristics (age and sex), distribution of etiologies according to different types of CLD, and changes of the etiologies over the last 10 years (2013–2022) were calculated for descriptive analysis of the study. Quantitative data (age of the CLD patients) were presented as mean ± SD, and qualitative data (sex, types of CLD, and distribution of etiology over the time) were presented in percentage. Comparison of etiologies over two time periods was also presented in percentage and analyzed by chi‐square test (*χ* ^2^). A *p* value < 0.05 was considered statistically significant. All data were analyzed by SPSS (Version 22.0, IBM Corp: Armonk, New York, United States). Data analyses were limited to available information only, and thus, a complete analysis of data of different etiological factors was not possible.

### 2.4. Diagnostic Criteria

Diagnosis of chronic hepatitis B was based on HBsAg positivity for more than 6 months with positive clinical and biochemical findings. Chronic hepatitis C was diagnosed by positive anti‐HCV with detectable HCV RNA. NAFLD was diagnosed based on ultrasonographic evidence of fatty liver in the absence of significant alcohol consumption, viral hepatitis, or other secondary causes, with or without elevated liver enzymes. AIH was diagnosed based on clinical features, elevated serum IgG levels, positive autoantibodies (ANA and ASMA), and/or liver biopsy findings when available, according to internationally accepted diagnostic criteria. Anti‐HBc (total)/occult HBV was defined as anti‐HBc positivity with negative HBsAg, with or without detectable HBV DNA. Diagnostic protocols remained consistent over the study period.

## 3. Results

The study included 4658 Bangladeshi patients diagnosed as CLD admitted into the Department of Hepatology at BSMMU from 2013 to 2022 with a mean age of 49.5 ± 13.8 years; most patients were aged 50–59 years (27.7%), with a male predominance (75.5%). Among various CLD patients, decompensated cirrhosis of the liver was the most prevalent (50.10%), followed by cirrhosis with HCC at 24.30%.

Etiological distribution of CLD reveals chronic HBV infection as the predominant cause, affecting 47.30% of patients, followed by cryptogenic liver disease (18.80%), chronic HCV infection (10.80%), whereas nonalcoholic fatty liver disease/nonalcoholic steatohepatitis (NAFLD/NASH) contributes to 5.20% during this period (Table 1).

**Table 1:** Etiology of chronic liver diseases (2013–2022) (*n* = 4658).

Etiology	Number of patients	Percentage (%)
HBV	2201	47.30
Cryptogenic	874	18.80
HCV	504	10.80
NBNC	463	9.90
NAFLD	240	5.20
Anti‐HBc (total)/occult HBV	198	4.30
Wilson′s disease	74	1.60
Alcohol	20	0.40
Autoimmune hepatitis (AIH)	17	0.40
Budd–Chiari syndrome (BCS)	16	0.30
Both HBV and HCV	16	0.30
Both NAFLD and anti‐HBc(T)	11	0.20
Both HCV and anti‐HBc (total)	10	0.20
Haemochromatosis	08	0.20
Both HBV and NAFLD	03	0.10
Both HCV and NAFLD	01	0.02
Both HBV and AIH	01	0.02
Both NAFLD and AIH	01	0.02
**Total**	4658	100

**Figure 2:**
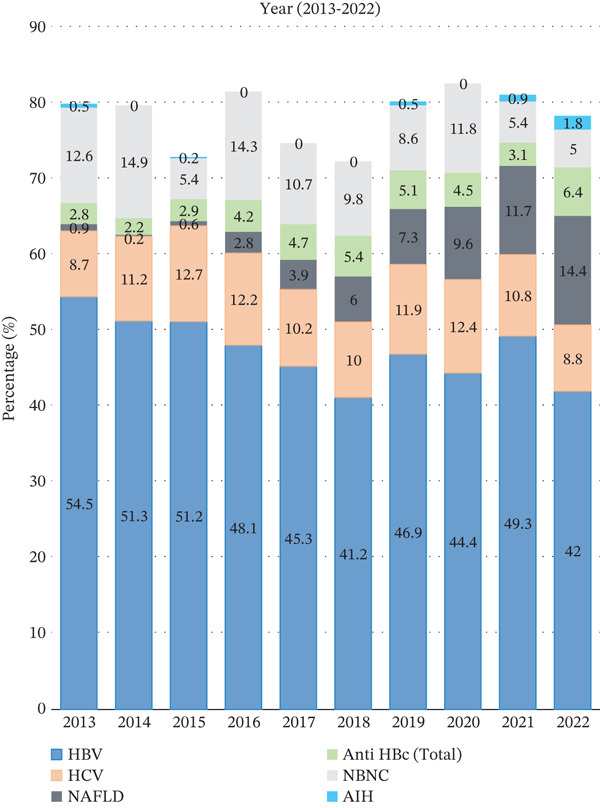
Year‐wise percentage distribution of major etiologies of chronic liver disease among admitted patients from 2013 to 2022 (*n* = 4658).

Year‐wise analysis demonstrated a decreasing trend in HBV prevalence (54.5% in 2013 to 42% in 2022); a relatively stable trend in HCV (8.7% in 2013 to 8.8% in 2022, with a slight increasing trend in the middle years) and increasing trend of NAFLD (0.9% in 2013 to 14.4% in 2022), anti‐HBc (total) or occult HBV (2.8% in 2013 to 6.4% in 2022), and AIH (0.5% in 2013 to 1.8% in 2022) (Table 2 and Figure 2).

**Table 2:** Comparison of etiologies of chronic liver disease between two time periods ([2013–2016] and [2017–2022]) (*n* = 4658).

Etiology	Time periods		*p*
	2013–2016	2017–2022	
HBV	1009 (51.1)	1192 (44.4)	**< 0.001**
HCV	224 (11.3)	280 (10.4)	**0.032**
NAFLD	24 (1.2)	216 (8.1)	**< 0.001**
BCS	3 (0.2)	13 (0.5)	0.055
Anti‐HBc (total)/occult HBV	61 (3.1)	137 (5.1)	**0.001**
Alcohol	10 (0.5)	10 (0.4)	0.492
Cryptogenic	362 (18.3)	512 (19.1)	0.506
NBNC	232 (11.7)	231 (8.6)	**< 0.001**
Both HBV & HCV	6 (0.3)	10 (0.4)	0.695
AIH	3 (0.2)	14 (0.5)	**0.038**
Haemochromatosis	2 (0.1)	6 (0.2)	0.318
Wilson′s disease	39 (2.0)	35 (1.3)	0.071
Both NAFLD and anti‐HBc (total)	0 (0.0)	11 (0.4)	—
Both HBV and NAFLD	0 (0.0)	3 (0.1)	—
Both HCV and anti‐HBc (total)	1 (0.1)	9 (0.3)	—
Both HCV and NAFLD	0 (0.0)	1 (0.0)	—
Both HBV and AIH	0 (0.0)	1 (0.0)	—
Both NAFLD and AIH	0 (0.0)	1 (0.0)	—
**Total**	1976 (100.0)	2682 (100.0)	—

*Note:* *p* value obtained by chi‐square test, *p* < 0.05 considered as a level of significant.

Bold values are statistically significant.

We have shown comparison of different etiologies of CLD between two time periods: (2013–2016) and (2017–2022) among 4658 patients. HBV was the predominant cause in both periods, comprising 51.1% in 2013–2016 and 44.4% in 2017–2022 (*p* < 0.001). Other significant etiologies were as follows: HCV was 11.3% in 2013–2016 and 10.4% in 2017–2022 (*p* = 0.032); NAFLD was 1.2% in 2013–2016 and 8.1% in 2017–2022 (*p* < 0.001); anti‐HBc (total) was 3.2% in 2013–2016 and 5.1% in 2017–2022 (*p* = 0.001); NBNC was 11.7% in 2013–2016 and 9.9% in 2017–2022 (*p* < 0.001); and AIH was 0.2% in 2013–2016 and 0.4% in 2017–2022 (*p* = 0.038). Notably, there was a significant decrease in the proportion of patients infected with HBV, HCV, and NBNC etiology from 2013–2016 to 2017–2022, whereas NAFLD, anti‐HBc (total), and AIH showed a significant increase. (Table 2).

Among the 4658 patients admitted to our department over the past decade, patients were disposed of in a wide variety of ways and death occurred in 5.90% of cases.

## 4. Discussion

Using the data of admitted patients in a tertiary level hospital of Bangladesh over the last 10 years, this study reports that the prevalence of HBV was declining, whereas the prevalence of HCV was almost flat. In contrast, the prevalence of NAFLD during the same period of time keeps increasing [16].

Regarding the etiology analysis of CLDs, we found that HBV is the most common cause, comprising 47.30% (Table 1), which is consistent with a previous study, where they found that HBV was the leading cause of CLD in the country, accounting for 61.15% cases of cirrhosis of liver [17]. Cryptogenic is the second most common cause (18.80%), followed by HCV (10.80%), which was the second most common cause in the previous study [17].

Although a steady increase in HCV was noted up to 2016 and recent reports suggest a fluctuating course followed by decline in the incidence of HCV since then. On the other hand, prevalence of NAFLD was steadily increasing over the last 10 years.

We compared the etiological distribution of CLD patients admitted into our department between two study periods: (2013–2016) and (2017–2022) over the last 10 years. There was a significant decrease in the proportion of patients with HBV infection from 2013–2016 to 2017–2022 (51.1%–44.4%, *p* < 0.001), HCV infection (from 11.3% in 2013–2016 to 10.4% in 2017–2022, *p* = 0.032); whereas NAFLD (from 1.2% in 2013–2016 to 8.1% in 2017–2022, *p* < 0.001) showed a significant increase (Table 2). All of these results were consistent with a previous study of United States. They have demonstrated the prevalence of CLD in the United States in the past 3 decades. A total of 58,731 adult patients from NHANES (1988–2016) were included. The prevalence of chronic hepatitis B and alcoholic liver disease remained static over the study period, and prevalence of chronic hepatitis C decreased almost twice. In contrast, the prevalence of NAFLD (by the United States Fatty Liver Index) has been increased from 20.0% (1988–1994) to 31.9% (2013–2016) (*p* < 0.0001) [16]. The etiological shift observed in this study, with a relative decline in HBV and a marked rise in NAFLD over the last decade, reflects broader global and regional liver disease patterns. Globally, NAFLD prevalence has been increasing substantially, with meta‐analytic evidence showing that overall prevalence rose from approximately 25% to near 38% in more recent years, and regional estimates in South Asia similarly high, underscoring a growing metabolic liver disease burden [18]. In contrast, chronic viral hepatitis—particularly HBV—continues to be a significant contributor to liver disease worldwide, but its relative impact has diminished in many settings where vaccination and prevention programs have been successfully implemented. The inclusion of hepatitis B vaccination into Bangladesh′s Expanded Programme on Immunization since 2003 has been associated with declining HBV prevalence in younger populations, although adult prevalence remains substantial [19]. In the South Asian region, systematic analyses report high NAFLD prevalence (around 27% in the general adult population), with even greater burden among individuals with metabolic comorbidities, indicating that metabolic risk factors are key drivers of liver disease in this population [20].

This study has several important limitations. First, it was a single‐center retrospective study conducted in a tertiary referral hospital, which may introduce referral bias and limit generalizability to the broader Bangladeshi population. However, as BSMMU is the largest tertiary hepatology referral center in the country, it receives patients from diverse geographic and socioeconomic backgrounds, partially mitigating this limitation. Second, data were extracted from a manually maintained hospital registry, which may have introduced information bias and missing data. Nevertheless, strict inclusion criteria and careful data verification were applied to minimize inaccuracies.

## 5. Conclusion

Over the last 10 years, the portrait of CLDs in Bangladesh has changed. This study indicates that although virus‐related CLD remain predominant, their relative proportion is declining compared with the rising burden of metabolic liver disease. With a successful immunization program against HBV, availability of DAAs (Direct acting antivirals) against HCV and with the gradual modernization of our lifestyle, this change is obvious over the recent years. People of our country are much more aware of HBV and HCV than before due to massive immunization and awareness programs, but they are indifferent about the increasing prevalence of NAFLD. With the result of this study, we can make people aware of the devastating outcome of this change and take strategic approaches to combat this huge burden.

## Author Contributions

Md. Hasan Shahriar, Avizit Sarker, and Md. Shahinul Alam conceived and designed the experiments. Md. Hasan Shahriar, Tapu Ghosh, and Md. Samsul Arafin performed the experiments. Md. Hasan Shahriar, Md. Shahinul Alam, and Avizit Sarker analyzed the data. Md. Hasan Shahriar, Tapu Ghosh, Md. Samsul Arafin, Md. Enamol Hoque, Md. Tanveer Rahman, Avizit Sarker, and Md. Shahinul Alam contributed to materials/analysis tools. Md. Hasan Shahriar, Md. Shahinul Alam, and Avizit Sarker wrote the paper.

## Funding

This study was supported by Bangabandhu Sheikh Mujib Medical University Thesis Research Grant under Memorial No. BSMMU/Thesis Research Grant/2024/71.

## Conflicts of Interest

The authors declare no conflicts of interest.

## Data Availability

The data used to support the findings of this study are available from the first author (hasanshahriardmc@gmail.com) upon request.
